# 

**Published:** 1908-01-15

**Authors:** 


### SUBJECT INDEX TO VOLUME LXII.

EVENT AND COMMENT.

Adenoids and the Dental Arch, 217.
Another Year, 597.
Cast Aluminum Dentures, 433.
Cast Inlays, 110.
Healthful Variety in Work, 595.
Indiana Semi-Centennial Celebration, 325.
Lost Time, 596.
Michigan State Meeting, 323.
Misdirected Energy, 55.
National Dental Association, 379.
New Constitution for National Dental Association, 541.
New Ohio Examining Board, 218.
New Work for Dental Societies, 487.
Northern Ohio Dental Association, 327.
Prescription Writing, 163.
Silk versus Calico, 1.
Stomatologic Section, A. M. A., 330.
The Dental Convention, 271.
The Oldest Dental Journal, 166.
Value of the Cast Inlay, 109.

ORIGINAL PAPERS.

Address, Presidents, O. E. Lanphear, 406.
A Plea for keener observation, more Conservatism, and a better understanding of which is the best Alloy, M. L. Ward, 381.
Avoiding Unnecessary Display in Bridge Work, W. A. Giffen, 5.
Care of Deciduous Teeth, O. W. White, 58.
Cause of Failures in the Use and Application of Porcelain, C. J. Lyons, 220.
Cause of Oxyphosphate Cements Disintegrating, J. O. Keller, 300.
Combination Fillings, J. J. Travis, 276.
Dental Progress as Indicated in Current Events, George C. Bowles, 544.
Dental Remedies, J. P. Buckley, 168
Dentifrices and Mouthwashes, Geo. W. Cook, 112.
Drugs, Abuses, Uses and Measurements, D. Stern, 185.
Gold Inlays, M. V. Hopkins, 229.
Management of Putrescent Pulps and Root Fillings, T. C. Reid, 144.
Metallic Posts to Fit Badly Decayed Root Canals, B. J. Cigrand, 434.
Oral Prophylaxis, Mabel M. Bennett 331.
Oral Surgery, C. H. Oakman, 28.
Orthodontia, its Significance to the General Practitioner, James D. Locke, 449.
Salient Points in the Histology of the Tooth, R. W. Bunting, 489.
School Children, Medical Examination of, Thos. B. Cooley, M. D. 614.
Some Things for Dentists to Think About, S. E. Dodson, 359.
Trials of Dentists Fifty Years Ago, Chas. H. Bartlett, 352.
Vaseline, W. G. Hamm, 86.

REPRINTS.

Cast Gold Inlays, E. E. Harcourt, 82.
Cause of Sensitive Dentine, C. F. Kabell, 530.
Comparative Qualities of Oxyphosphates, R. B. Fuller, 294.
Dental Friendships, C. P. Elder, 579.
Dental Inspection and Treatment of School Children, R. M. Capon, 139
Inlay Veneer, W. C. Davis, 39.
Interstitial Gingivitis, E. C. Briggs, 499.
Intra Alveolar Injection for Anesthesia, F. L. Fossume, 528.
Leucodescent Light for Control of Pain, F. E. Raiche, 364.
Mouth Breathing its Cause and Consequences, Woods Hutchinson, 248.
Novocaine Studies, R. H. Reithmuller, 515.

Observations on the Cause and Prevention of Dental Caries, J. Sim Wallace, 599.
Pulp Reunion, A. H. Fuller, 87.
Pyorrhoea Alveolaris and its Treatment, A. J. Macdonagh, 456.
Quackery in English Dentistry, its Cause, R. S. Grant, 147.
Report of Committee on Alloys, 467.
Spinal Anesthesia, Jno. S. Marshall, 193.
Sulpho-Carbolic Acid, E. MaWhinney, 308.
Tin and Gold, C. E. Kells, 577.
Tumors of the Jaw, M. Schamberg, 145.
Value of Diagnosis in Filling Teeth, J. V. Cowzett, 416.

INDENTS.

Acetanilid Compound, 185.
Adhesive Wax Trial Plates, 370.
Administering Gas, 151.
Amalgam and Cement Fillings, 101.
Amalgam Inlays, 635.
An Accurate and Safe Way of Applying Silver Salts, 587.
A New Crown, 472.
Anodyne Remedy, 183.
Anodyne Paste, 185.
Antacid Tooth Powder, 121.
Antiseptic Spray for Teeth and Gums 202, 369.
Application for Sensitive Dentine, 98.
Antiseptic Powder for Pyorrhoea, 474.
Arsenical Necrosis, 100.
Articulating Paper, 150.
Artificial Patina on Brass, 202.
Assistants, 629.
Astringent Solution, 184.
Bite and Face Bow, 312,
Blind Dentists, 313.
British Odontological Society, 150.
Canadian Oral Prophylactic Association, 204.
Canal Treatment, 369.
Careless Use of Bands and Ligatures in Regulating, 537.
Cast Inlays, 50, 311.
Catarrh, 150.
Cement Solution in Acid Solution, 477.
Chloretone in Post Operative Nausea and Vomiting, 97.
Cleaning Impression Trays, 150.
Consolidation of Registry Boards, 203.
Contouring Artificial Dentures, 90.
Crown Helps, 584.
Crown and Bridge Work of the Future, 48.
Curing Pyorrhoea, 102.
Death from Chloroform, 152.
Death After Nitrous Oxide, 154.
Dental Manufacturers' Exhibit, 213.
Dental Surgery in Antiquity, 158.
Dentistry in the French Army, 154.
Dentists Earning Capacity, 201.
Dentists Honor Dr. H. M. Reid, 470.
Devitalizing Paste, 183,
Diet, Health and Fletcherism, 264.
Difficult Impressions, 370.
Dignity of Prosthesis, 372.
Dilute Hydrochloric Acid in Amalgamating Alloys, 94.
Disease in the Public Schools, 203.
Early Crown Work, 151.
Electric Wire Joining, 633.
Electricity as an Anesthetic, 589.
Enlarging Pulp Canals, 630.
Enthusiasm tempered with Judgement, 266.
Erosion and Sensitiveness, 586.
Evans Will Contest Settled, 45.
Facts for Patients, 375.
Failure in Tooth Extraction, 90.
Finishing Gold Inlays, 627.
Fixed and Removable Bridges, 47.
Flux for Base Metals, 203.
German Dental Decision, 591.
Gold and Amalgam Combination, 95
Guthymol, 371.
Hopeless Pyorrhoea Teeth, 96.
How Much Mercury, 374.
How to Stop Bleeding after Extraction, 475.
Hydrofluoric Acid, 631.
Immediate Root Filling, 628.
Impression Varnish, 202.
Inlay Model Smoking History, 211.
Instruction in Oral Sanitation, 91.
Investment Material, 151, 626.

Iron Preparations, Action on Teeth, 206.
Lead Points as Root Fillings, 153.
Liniment, 183, 184.
Liquor Antisepticus, 182.
Local Anesthetic, 153, 184, 510.
Local Anesthetic for Pyorrhoea, 151.
Malocclusion, 628.
Matrices, 632.
Mechanical Dentists, 315.
Medical Graduates of 1908, 631.
Metallic Investment for Delicate Appliances, 626.
Mixing Investment for Inlays, 582.
Modified Eucalyptol, 183.
Modified Phenol, 185.
Mouth Washes, 632.
Neuralgia, 184.
Nitrous Oxide in Major Operations, 372.
Non-Cohesive Foil, 152.
Non-Inflammable Celluloid, 372.
Novocaine Formula, 517, 526.
Neuralgia, 184, 185.
Objection to Immediate Root Filling 370
Occlusion, 590.
Oil Polish in Prophylaxis, 479.
Opening Abscess Cavities, 371.
Oral Prophylaxis, 373.
Orthodontia Adenoids, 93.
Orthodontia Specialists, 587.
Partial Impressions, 91.
Patience, 424.
Personal Appearance, 47.
Pharmacopeal Drugs, 157.
Poison in Cigarettes, 91.
Porcelain Epitome, 316.
Porcelain in its Place 628.
Porcelain Mixed with Alcohol, 537.
Porcelain versus Silicates, 629.
Post Extraction Treatment, 99.
Posts for Bicuspids, 627.
Preparation and Uses of Fluxes, 423.
Preventing Cement and Gutta-Percha Sticking to Instruments, 538.
Prevention of Tooth Decay, 153.
Primitive Dentistry, 150.
Progress in Partial Dentures, 318.
Prophylaxis Abrasions, 314.
Proprietary Remedies, 155.
Proteol, 152.
Protection of Dentine against Specific Infection, 103.
Pulp Anesthesia, 150.
Pulp Removal, 153.
Putrescent Pulps, 184.
Pyorrhoea Treatment, 630.
Quack Medical Advertisement Evils, 209.
Refrigeration without Pain, 626.
Relative Solubilities of Cements, 93.
Retaining Teeth by Means of Inlays, 93.
Removing Morbid Growths from Cavities, 43.
Removing Bridges, 537.
Root Bands, 44.
Root Filling for Deciduous Teeth, 46.
Sacrificing Natural Crowns, 45.
Secret Formula, 206.
Securing Patients, 473.
Separating Teeth, 588.
Serum Treatment, 582.
Setting Crowns with Gutta-Percha, 43.
Short Cut Methods, 96.
Silicate Cements, 94.
Sodium Dioxide, 592.
Solidified Formaldehyde with Cassia and Sodium Glycerate Base, 585.
Specialization, 207.
Stability of Artificial Dentures, 100.
Sterilizing Instruments, 634.
Stickey Wax, 583.
Stove Polish on Inlay Models, 626.
Talbott's Fomula, 184.
Tannic Acid for Pyorrhoea, 422.
Temperaments and Occlusion of Artificial Teeth, 373.
Thorough Mastication may be overdone, 585.
Tin Foil in Plate Work, 634.
Toxic Effects of Quinosol, Cresol and Lysol, 205.
Treatment of Root Canals, 98.
Treatment of Pyorrhoea, 426, 471.
To Cap a Badly Broken Molar, 95.
To Insert an Amalgam Filling as an Inlay, 92.
To Mount Carborundum Stones, 97.
To Remove Rust from Instruments, 470.
Tooth Powder, 129.

Use of Blue Light, 92.
Unsolved Problems in Bridge Work, 90.
Varnish for Impressions, 369.
What shall be our attitude towards a child with irregular teeth and poor parents, 263.
Why it adheres, 422.
Wire Connection for Lower Partial Dentures, 44.
Zinc Chloride, 633.

AUTOGRAPHIC.

Allen, A. B., 152.
Allen, G. S., 467.
Ames, W. V. B., 315.
Arnold, L. H., 586.
Bailey, R. E., 77.
Baker, T. T., 537.
Barbour, F. W., 628.
Bartlett, C. H., 352.
Becker, S., 287.
Bennett, Mabel M., 133, 352.
Beshoar, J. M., 101.
Bevan, A. D., 372.
Black, G. V., 150, 375, 533.
Blackmarr, C. B., 361.
Blake, Mary E., 98.
Bodecker, H. W. C., 635.
Bowles, G. C., 13, 68, 127, 132, 244, 410, 544, 575.
Brady, W. J., 537.
Brophy, T. W., 371, 374,
Brophy, R. C., 444.
Briggs, E. C., 499, 514.
Brunton, G., 151.
Buckley, 100, 168, 633.
Bunting, R. W., 24, 292, 489, 497, 571.
Burke, Geo., 67, 72, 130, 346.
Buttrick, T. R., 131, 247, 396.
Byram, J. Q., 538.
Caple, A. Z., 150.
Capon, R. M., 139.
Capon, W. A., 628.
Case, C. S., 482.
Catterell, A. J., 370.
Cigrand, B. J., 413, 434, 446.
Collins, T. J., 121.
Cooley, T. B., 614.
Cowzett, J. V., 202, 369, 416, 589, 590.
Cook, G. W., 112, 134.
Cooke, A. R., 424.
Carley, Dr., 92.
Crouse, J. N., 267.
Dolbey, W. C., 473.
Davis, W. C., 39.
Day, E. F., 151.
Dodson, S. E., 359, 363.
Dittmar, G. W., 627.
Dumas, W. A., 129.
Elder, C. P., 579.
Ferris, H. C., 46.
Flanagan, A. J., 587.
Fossume, F. L., 528.
Fuller, A. H., 87.
Giffen, W. A., 5, 27, 71.
Goslee, H. J., 47, 49, 90, 321.
Graham, Don. M., 132, 291, 347, 566.
Grant, R. S., 147.
Hall, J. S., 67, 73.
Hamm, W. G., 86, 584.
Hamlet, Dr., 627.
Harcourt, E. E., 82.
Haskins, G. W., 152.
Hind, Ella M., 630.
Hoff, N. S., 25, 74, 122.
Hoff, N. S., 25, 74, 122, 338, 344, 361 410, 441, 513, 514.
Hungerford, C. L., 103.
Hunt, G. E., 94.
Hutchinson, Jr., R. G., 472.
Hutchinson, W., 248.
Howe, J. M., 467.
House, J. W., 241.
Hopkins, M. V., 229, 247.
Jones, W. H., 369.
Johnson, C. N., 210, 264, 312, 425.
Kabell, C. F., 530.
Keller, J. O., 300.
Kells, C. E., 577.
Kettig, E. M., 100.
King, O. N., 474.
Kleinsorgen, Dr., 480.
Kner, M., 422.
Land, C. H., 289, 399, 443.
Lanphear, O. E., 406.
Leach, T. A., 151, 202, 369.
Lee, A. P., 212.
Le Gro, A. L., 125, 245.
Locke, J. D., 449.
Loeffler, E. T., 77, 498.
Lyons, C. J., 220.
Marshall, J. S., 193.

MaWhinney, E., 308.
Maxfield, G. A., 95.
Macdonagh, A. J., 456.
McCracken, Dr., 538.
McMillan, H. W., 375.
Merritt, A. H., 158, 467.
Morris, R. T., 635.
Myers, C. G., 98.
Noel, L. G., 43.
Nies, Dr., 627.
Noble, W. C., 203, 629.
North, G., 673.
Oakman, C. H., 28.
Ottolengui, R., 583, 631.
Parshall, H. E., 64.
Patterson, J. D., 97, 629.
Peck, A. H., 514.
Perry, S. G., 632.
Platt, Dr., 99.
Powell, T. E., 318.
Prinz, H., 209.
Prothero, J. H., 90, 313.
Prwyn, C. P., 95.
Raiche, F. E., 364.
Reid, T. C., 414.
Reithmuller, R. H., 515.
Rhein, M. L., 512.
Roach, F. E., 44.
Rogers, G. P., 350.
Root, J. P., 102.
Rose, Harry, 371.
Royce, A. E., 628.
Runyan, A. F., 362, 440.
Schaeffer, Dr., 21.
Schamberg, M., 145.
Smith, C. L., 91.
Solbrig, O., 96.
Soule, E. M., 593.
Spalding, E. B., 67, 237, 345, 349, 487.
Spring, J. F., 77.
Stern, David, 185.
Stewart, J. B., 106.
Straith, S. S., 350.
Strang, Dr., 588.
Taggart, W. H., 150, 316.
Travis, J. J., 23, 25, 276, 293.
Trueman, W. H., 167, 626.
Tuller, R. B., 294, 314.
Vance, Dr., 92.
Van Woert, Dr., 372.
Ward, M. L., 131, 242, 381, 403, 412, 561.
Wallace, J. S., 599.
Watkins, S. C. G., 634.
Watson, M. T., 66, 71.
Weeks, Thos., 51.
Wheeler, H. L., 157.
White, O. W., 58, 79.
Whitslar, W. H., 428.
Williams, F. E., 362.
Wilson, G. H., 96, 330.
Wood, C. P., 22, 341, 345, 573.
Zacharais, C., 371.

### ANNOUNCEMENTS.

American Dental Degree Recognized, 52.
American Medical Association, 431.
American Society of Orthodontists, 539.
Army Oral Hygiene, 638.
Black's Operative Dentistry, 533.
Byram's Principles and Practice of Porcelain, 485.
Cadillac, Mich., Dental Society, 323.
Canadian Dental Association, 377.
Cases Dental Orthopedia, 482.
Cincinnati Dental College Graduates 377.
Dental Manufacturer's Exhibit, 430.
Dental Graduates for 1908, 638.
Detroit College Graduates, 378.
District of Columbia Examiners, 639
Examination for Army Positions, 593.
Gray's Anatomy, 430.
Illinois State Dental Society, 215, 429.
Indiana Dental Society Clinicians, 268.
Indiana Examiners, 593, 637.
Institute of Dental Pedagogy, 201.
Interstate Dental Fraternity, 322.
Jamestown Dental Convention, 482.
Kentucky Dental Association, 214.
Massachussetts Board of Registration, 539.
Michigan Eighth District Society, 324
Michigan First District Dental Society, 53.
Michigan Second District Dental Society, 105.
Michigan State Dental Society, 106, 162.

Missouri Dental Association, 161, 428.
Montana Examiners, 322.
National Dental Association, 321, 376, 481, 637.
N. D. A. History Committee, 429.
New Editor on BRIEF, 54.
New Jersey Board of Examiners and Registration, 105, 538.
Northern Ohio Dental Association, 161, 214.
Ohio College of Dental Surgery Graduates, 377.
Ohio State Board of Registration, 270, 539.
Ohio State Dental Society, 53, 593.
Pennsylvania State Dental Society, 323,
Ranson & Randolph Co. Reorganized, 201.
Semi-Centennial of Indiana Dental Association, 105.
Southern Branch National Dental Association, 212.
South Dakota Examiners, 323.
St. Louis Dental College Clinic, 161.
University of Michigan Graduates, 378.
Virginia Dental Association, 215, 324.
West Virginia Examiners, 162.

OBITUARY.

Corbin, G. E., 481.
Fillebrown, Thos., 199.
Kerr, Jno., 539.
Northup, A. L., 594.
Randolph, J. F., 52.
Ransom, J. R. B., 52

### ANNOUNCEMENTS.

New Book On Operative Dentistry.—The Medico-Dental Publishing Co., of Chicago, announces for publication a new work on Operative Dentistry by Dr. G. V. Black. The work will be issued sometime in March, and will be in the form of a two volume royal octavo style with large type, printed on extra paper, and contain about 800 pages of reading matter and 600 illustrations. Dr. Black has been teaching Operative Dentistry for many years and has done more scientific investigation of technical subjects than any other man. The text as well as the illustrations will be new and characteristic of the man. It will be a one man book, therefore consecutive and more valuable as a text work than the so-called "systems." We have no doubt but it will meet a large sale, and will for many years be the standard work on this subject. We wait its appearance with pleasant anticipations.

DEATH OF J. R. B. RANSOM AND I. F. RANDOLPH.—The dental profession will be shocked to learn of the sudden deaths of these two gentlemen who have become widely known to the profession, as the organizers of the Dental Supply house of The Ransom & Randolph Co. of Toledo, Ohio, and publishers of the *Dental Summary*. Mr. Ransom died in San Francisco, California, December 17th, 1907, and Mr. Randolph three days later in Toledo, Ohio. They were both honorable and capable business men, and their house stood well commercially and with the profession for more than a quarter of a century. The business will be carried on the the same as before as it is incorporated and for several recent years the younger members of the firm Messrs. Bigelow and Crandall have been more active in its conduct than the original founders.

THE AMERICAN DENTAL DEGREE IS OFFICIALLY RECOGNIZED IN GERMANY.—After a long legal contest one of the highest German law courts has decided that American dentists holding diplomas from reputable American Dental

Colleges can use their professional titles of Doctor of Dental Surgery. This does not imply that they can register for practice on the same standing as the German Zahnarzt; but is a distinct gain, as the German dentists have been for years trying in every way to discredit the American degree and have it outlawed.

---

MICHIGAN FIRST DISTRICT DENTAL SOCIETY.—The Detroit Dental Society is no more, by its action it has now become the Michigan First District Dental Society. This is the first organization under the new plan of districting the state and affiliating all societies with the State Society. The First District Society will take in Wayne County and parts of the adjoining counties. It will be the largest and strongest District in the state and is therefore entitled to be named the First. We trust that it will also make its claim to the name worthy by doing better and more good professional work than any other District.

---

CLINIC OF MICHIGAN FIRST DISTRICT DENTAL SOCIETY. —The Annual Clinic will be held at Detroit, Saturday, Feb. 15th, 1908. All ethical dentists are most cordially invited to attend. A good program is assured.

DON M. GRAHAM, D. D.S., *Secretary.*

---

OHIO STATE DENTAL SOCIETY—The Annual meeting was held in Columbus December 3, 4, and 5, 1907, at the Great Southern Hotel. This society always has a good meeting. This year it was exceptionally so. There was a large attendance. The papers were well considered and ably discussed; the clinics were numerous, interesting and instructive; the exhibits of dealers and manufacturers were many and so enticing that they divided the honors fairly with the clinics. There is but one criticism that we want to make on this meeting and it applies to those that have recently preceded it. The hall where the papers are read and discussed is wholly inadequate and so uncomfortable that this the most important part of the meeting suffers greatly. The accommodations for the clinicians and exhibitors are very good indeed. We hope the management will next year secure a suitable hall for the reading of papers etc. We are sure it will pay well for any expense or other inconvenience involved. The society elected Dr. C. I. Keeley of Hamilton, President, Dr. W. H. Whitslar of Cleveland, 1st Vice-President, M. H. Fletcher, Cincinnati, 2nd Vice-President, Dr. F. R. Chapman of Columbus, Secretary, and Dr. W. A. Price of Cleveland, Treasurer.

New Editor On Dental Brief.—Dr. Wm. H. Trueman has succeeded Mrs. J. M. Walker as Editor of "Practical Points" in the Dental Brief. Dr. Trueman is an old editor and a book lover and we have no doubt he will prove a worthy successor of that remarkable woman, who has for so many years read our literature with so much care and discernment that she made her department one of the greatest value, and in her death the profession has lost a devoted friend.

